# Mental health services in the Philippines

**DOI:** 10.1192/bji.2018.34

**Published:** 2019-08

**Authors:** John Lally, John Tully, Rene Samaniego

**Affiliations:** 1 Psychiatrist and Clinical Lecturer, Department of Psychosis Studies, Institute of Psychiatry, Psychology and Neuroscience, King's College London, UK. Email: john.lally@kcl.ac.uk; 2 Psychiatrist and Clinical Lecturer, Department of Psychiatry, Royal College of Surgeons in Ireland, Beaumont Hospital, Ireland; 3 Psychiatrist and Clinical Lecturer, Department of Psychiatry, St Vincent's Hospital Fairview, Ireland; 4 Psychiatrist and Clinical Lecturer, Department of Forensic and Neurodevelopmental Sciences, Institute of Psychiatry, Psychology and Neuroscience, Kings College London, UK; 5 Psychiatrist, Section of Psychiatry, Department of Neurosciences, Makati Medical Center, the Philippines

**Keywords:** Asia, psychiatry, lower-middle income, education and training

## Abstract

National information on mental health services in the Philippines indicates that there are substantial gaps and inconsistencies in the delivery of mental healthcare. The recently enacted Mental Health Act legislation provides a platform for the delivery of comprehensive and integrated mental health services. However, there remain many challenges in the provision of accessible and affordable mental healthcare.

The Philippines is an autonomous republic located in the Western Pacific, with a population of over 100 million and a large diaspora of approximately 10 million people. It is the 12th most densely populated country in the world. The Philippines is an archipelago of over 7000 islands, with the majority of the population living on the largest islands of Luzon (in which the capital Manila is located), Visayas and Mindanao. The country stretches towards Taiwan in the north and to Indonesia and Brunei in the south, with the Pacific Ocean to the east, and the South China Sea divides it from mainland South East Asia.

Most (90%) of the population is Christian, with 80% Roman Catholic, and approximately 5% are Muslim. Filipino is the official language, although English (the second language) is widely spoken.

The Philippines is classified as a lower-middle-income country (defined as a gross national income per capita of between $1006 and $3955), based on the 2017 per capita income statistics by the World Bank (https://data.worldbank.org). Economic improvements have been evident over the past decade.

The Philippines attained full independence in 1946 after being colonised and occupied by foreign powers since 1545: first by Spain, then by the US and finally by Japan during the Second World War. This colonial history contributed to the development of a unique Filipino culture, which also includes ancient and contemporary Asian influences. However, it remains a country poorly understood in the West; it is often viewed as an apparent anomaly in the region due to its belated achievement of independence and its uniqueness as a Christian-majority country in Asia.

## Mental health services

The Philippines has recently passed its first Mental Health Act (Republic Act no. 11036). The Act seeks to establish access to comprehensive and integrated mental health services, while protecting the rights of people with mental disorders and their family members (Lally *et al*, 2019). However, mental health remains poorly resourced: only 3–5% of the total health budget is spent on mental health, and 70% of this is spent on hospital care (WHO & Department of Health, 2006).

Accordingly, the majority of mental healthcare is provided in hospital settings and there are underdeveloped community mental health services. The National Center for Mental Health was previously estimated to account for 67% of the available psychiatric beds nationally (Conde, 2004). More recent data indicate that there are 1.08 mental health beds in general hospitals and 4.95 beds in psychiatric hospitals per 100 000 of the population (WHO, 2014). There are 46 out-patient facilities (0.05/100 000 population) and 4 community residential facilities (0.02/100 000) (WHO, 2014). There are only two tertiary care psychiatric hospitals: the National Center for Mental Health in Mandaluyong City, Metro Manila (4200 beds) and the Mariveles Mental Hospital in Bataan, Luzon (500 beds). There are 12 smaller satellite hospitals affiliated with the National Center for Mental Health which are located throughout the country. Overcrowding, poorly functioning units, chronic staff shortages and funding constraints are ongoing problems, particularly in peripheral facilities. There are no dedicated forensic hospitals, although forensic beds are located at the National Center for Mental Health.

## Mental health staff

There is 1 doctor for every 80 000 Filipinos (WHO & Department of Health, 2012); the emigration of trained specialists to other countries, particularly English-speaking countries, contributes to this scarcity. This shortage is magnified in psychiatry where, nationally, there are a little over 500 psychiatrists in practice. The ratio of mental health workers per population in the Philippines is low, at 2–3 per 100 000 population (WHO & Department of Health, 2006). This ratio is lower than in other Western Pacific Rim countries with similar economic status, for example Malaysia (4.9 mental health workers per 100 000 population) and Indonesia (3.1 per 100 000 population). Data indicate that there are 0.52 psychiatrists (Isaac *et al*, 2018) and 0.07 psychologists per 100 000 inhabitants, and 0.49 mental health nurses per 100 000 of the population (a reduction from 0.72 per 100 000 in 2011) (WHO, 2014).

Together, these figures equate to a severe shortage of mental health specialists in the Philippines. This is further illuminated when compared with the World Health Organization (WHO)-recommended global target of 10 psychiatrists per 100 000 population. Further, the majority of psychiatrists work in for-profit services or private practices and are mainly based in the major urban areas, particularly in the capital region known as Metro Manila.

## The burden of mental disorders in the Philippines

There is little epidemiological evidence on mental disorders in the Philippines; however, some important data are available. For example, 14% of a population of 1.4 million Filipinos with disabilities were identified to have a mental disorder (Philippines Statistics Authority, 2010). The National Statistics Office identified that mental illness is the third most prevalent form of morbidity, however the finding that only 88 cases of mental health problems were reported for every 100 000 of the population (DOH, 2005) is likely an underestimate of the true extent of these issues.

The 2005 WHO World Health Survey in the Philippines identified that, of 10 075 participants, 0.4% had a diagnosis of schizophrenia and 14.5% had a diagnosis of depression. Of those with a diagnosis of schizophrenia, 33.2% had received treatment or screening in the past 2 weeks, compared with 14% of those with a diagnosis of depression. Recent data from the Philippine Health Information System on Mental Health identified that (from 14 public and private hospitals surveyed from 2014 to 2016) 42% of the 2562 surveyed patients were treated for schizophrenia.

Between 1984 and 2005, estimates for the incidence of suicide in the Philippines have increased from 0.23 to 3.59 per 100 000 in males, and from 0.12 to 1.09 per 100 000 in females (Redaniel *et al*, 2011). The most recent data from 2016 identified an overall suicide rate of 3.2/100 000, with a higher rate in males (4.3/100 000) than females (2.0/100 000) (WHO, 2018).

## Access to treatment

Prohibitive economic conditions and the inaccessibility of mental health services limit access to mental healthcare in the Philippines. Further, perceived or internalised stigma has been shown to be a barrier to help-seeking behaviour in Filipinos (Tuliao & Velasquez, 2014), just as is the case in Western populations (Lally *et al*, 2013). There is a cultural drive to ‘save face’ when there is a threat to or loss of one's social position, and as such Filipinos may have difficulty in admitting to mental health problems or seeking help. There is a strong sense of family in the Philippines and so, when problems are thought to be socially related, Filipinos will turn to family and peer networks before seeking medical help (Tuliao, 2014).

There are little data on prescription rates and the use of psychotropic medications in treating mental disorders. The 2005 WHO Health Survey indicated that only a third of people with a diagnosis of schizophrenia were receiving treatment or screening (although antipsychotic medication was not specified as the treatment).

There is a national Department of Health Medication Access Program for Mental Health that carries a central list of essential medications, which are shown in Box 1. These medications are available at all service levels, but funding issues limit patient access, particularly access to newer medications. The most commonly used antipsychotics in clinical practice are chlorpromazine and haloperidol; escitalopram and fluoxetine are the most commonly used antidepressants.

Box 1. The Philippines Department of Health Medication Access Program for Mental Health list of essential psychotropic medications

(a) First-generation/typical antipsychotics → chlorpromazine, haloperidol (oral and long-acting injectable), fluphenazine decanoate
(b) Second-generation/atypical antipsychotics → clozapine, olanzapine, quetiapine, risperidone
(c) Antidepressants → fluoxetine, sertraline, escitalopram
(d) Mood stabilisers → lithium carbonate, valproic acid, carbamazepine, lamotrigine
(e) Anticholinergics → biperiden, diphenhydramine
(f) Benzodiazepine → clonazepam
(g) Cholinesterase inhibitor → donepezil
(h) NMDA receptor antagonist → memantine

## Psychiatry training

There are currently 47 accredited medical schools in the Philippines. Psychiatry is a recognised core part of the medical curriculum, which is generally 4 years long. The average time allotted for the psychiatry module is 2 weeks, which incorporates teaching by lectures and clinical exposure. There are 13 postgraduate psychiatry training institutions, with 8 of them based in the Metro Manila region, including 1 based at the National Center for Mental Health. The others are generally located at regional or tertiary hospitals. Only two of the postgraduate training programmes offer 2-year fellowships in psychiatric subspecialties such as child and adolescent psychiatry, consultation–liaison psychiatry, community psychiatry and addiction psychiatry.

Postgraduate residency training is generally a 3- or 4-year programme, depending on the institution. The individual training institution is responsible for designing the training programme, with core competencies acquired in line with international standards. Trainees have the opportunity to spend 3 months in neurology and 2 months in an internal medicine department. All institutions conduct written examinations and Objective Structured Clinical Examinations (OSCEs). Upon completion of residency training, an exit examination is performed with written and OSCE components, following which the title Diplomate of the Specialty Board of Philippine Psychiatry is awarded to the candidates who qualify.

Despite these structures, psychiatry remains a less popular specialty for medical graduates in the Philippines, and the numbers being trained are inadequate to meet a growing need.

## Conclusions

Mental healthcare in the Philippines faces continued challenges including underinvestment, lack of mental health professionals and underdeveloped community mental health services. Although the recent Mental Health Act legislation has – for the first time – provided a legal framework for the delivery of comprehensive mental healthcare, economic restrictions preventing people from accessing mental healthcare should be considered to enable the population to equitably access appropriate care when required. Increased investment is urgently needed to improve the training and recruitment of psychiatrists, nurses, psychologists, social workers and other multidisciplinary team members, particularly as large numbers of skilled professionals continue to emigrate.
